# Bacterial Subcellular Architecture, Structural Epistasis, and Antibiotic Resistance

**DOI:** 10.3390/biology12050640

**Published:** 2023-04-23

**Authors:** Fernando Baquero, José-Luis Martínez, Alvaro Sánchez, Miguel D. Fernández-de-Bobadilla, Alvaro San-Millán, Jerónimo Rodríguez-Beltrán

**Affiliations:** 1 Department of Microbiology, Ramón y Cajal University Hospital, Ramón y Cajal Institute for Health Research (IRYCIS), 28034 Madrid, Spain; migueldiez@outlook.com (M.D.F.-d.-B.); jeronimo.rodriguez.beltran@gmail.com (J.R.-B.); 2 CIBER en Epidemiología y Salud Pública (CIBERESP), 28034 Madrid, Spain; 3 Centro Nacional de Biotecnología, CSIC, 28049 Madrid, Spain; jlmtnez@cnb.csic.es (J.-L.M.); alvarosanchez@gmail.com (A.S.); alvsanmillan@gmail.com (A.S.-M.); 4 CIBER en Enfermedades Infecciosas (CIBERINFECT), 28034 Madrid, Spain

**Keywords:** bacterial Gram-negative subcellular architecture, structural epistasis, cellular shape and volume, antibiotic mode of action, antibiotic resistance

## Abstract

**Simple Summary:**

The concept of “structural epistasis” expresses the emergence of new phenotypes which are not based on changes in the products and functions of genes, but on the changes in the physical–mechanical interactions between biological structural pieces and components of the bacterial cell architecture. These interactions are fostered by primary physical changes in the shape and size of the pieces or in spatial (topological) alterations driven by changes in their quantity or local density of the cell compartments, and might have consequences on antibiotic resistance phenotypes.

**Abstract:**

Epistasis refers to the way in which genetic interactions between some genetic loci affect phenotypes and fitness. In this study, we propose the concept of “structural epistasis” to emphasize the role of the variable physical interactions between molecules located in particular spaces inside the bacterial cell in the emergence of novel phenotypes. The architecture of the bacterial cell (typically Gram-negative), which consists of concentrical layers of membranes, particles, and molecules with differing configurations and densities (from the outer membrane to the nucleoid) determines and is in turn determined by the cell shape and size, depending on the growth phases, exposure to toxic conditions, stress responses, and the bacterial environment. Antibiotics change the bacterial cell’s internal molecular topology, producing unexpected interactions among molecules. In contrast, changes in shape and size may alter antibiotic action. The mechanisms of antibiotic resistance (and their vectors, as mobile genetic elements) also influence molecular connectivity in the bacterial cell and can produce unexpected phenotypes, influencing the action of other antimicrobial agents.

## 1. Introduction

Etymologically, the term “epistasis” means the “act of stopping” (any “on–off” action) and can be applied to the case where a mutation influences the effect of other mutations, in a specific or unspecific and less evolutionarily stable manner [1,2]. Epistasis is also a phenomenon in which one or more genes influence the function of others. The related term “epigenetics” refers to studies “above the gene” and the heritable (reproducible) changes in gene function that cannot be explained by DNA sequence mutations [3]. In both cases, the organism exhibits a function that cannot be fully explained by the sequence of a single gene. The most classic cases of epistasis rely on the interconnection of regulatory networks. For instance, a mutation that alters the concentration of a metabolite that regulates the expression of other metabolic routes may deeply alter the bacterial physiology, thereby modifying the activities of other genes/proteins. Examples of this include the modification of the physiology of antibiotic-resistant mutants [4,5] or the various evolutionary pathways toward resistance that bacteria with differing genomic backgrounds can follow, which is an example of historical contingency [6]. However, these regulatory and functional alterations can have an important structural role, a feature largely underexplored.

In general, and in the abovementioned epistatic examples, a phenotype is the result of the interconnected functions of an ensemble of genes (not necessarily linked), as in the case of an operon encoding for antibiotic resistance, or in biosynthetic pathways. In the last case, however, the function of each involved gene is autonomous and specific; for instance, encoding an enzyme needed for a particular biochemical reaction. The operational molecules involved in epistasis are the products of interacting genes: proteins interact with other proteins, small molecules or nucleic acids. In *Escherichia coli*, the concentration of these macromolecules can exceed 300 g/L, occupying approximately 30% of the cell volume [7,8].

In population genetics, epistasis refers to how genetic interactions between some loci affect phenotypes and fitness [9]. The concept of structural epistasis helps explain the emergence of new phenotypes that are not based on changes in gene function, but on the physical–mechanical interactions between the biological “structural pieces” or components of the cell. These interactions result from primary physical changes in the piece’s shape and size, or in the spatial (topological) alterations driven by changes in their quantity or local density. This might result in spatial heterogeneities leading to various cell-to-cell physical interactions, that also have consequences in microbial cell biology. The term “cell topology” is employed in studies on the structure of tissues, where spatial heterogeneity might produce differing biological outcomes [10]. Starting about one decade ago, new technologies have become available to study intracellular topology, as multi-scale fluorescence cross-correlation spectroscopy [11].

A bacterial cell is composed of complex physical multimolecular objects, which include: (1) ball-shaped complex structures, such as ribosomes, supercoiled DNA in the chromosome (forming a nucleoid) or in bacterial plasmids; (2) lamellar structures, such as the cell wall, membranes or capsules; (3) elongated structures, such as fimbriae and flagella; (4) complex-shaped functional organelles, ranging from low complexity, such as porins, to high complexity, such as extrusion pumps, needle-like protein complexes in the type III secretion system, or trans-envelope flagella machines; and (5) ball-shaped inclusion bodies, typically water-insoluble protein aggregates or condensates, glycogen poly-beta-hydroxybutyrate granules, or cyclic polyphosphate inclusions. These complex structures in the bacterial cytoplasm, and the molecular crowded cytoplasm itself, have inherent physical properties, such as viscoelasticity [12] or electric charge, which affect their function [13]. Molecules have both a physical and chemical dimension. For instance, the interaction and function of proteins depends on their tertiary structure, the three-dimensional arrangement (folding) of its polypeptide chain in space, and on their four-dimensional (protein-assembly in multimeric proteins) and fifth-dimensional (quinary structure, see below) structures, ensuring the interactions between macromolecules that organize the interior of the cell.

Similarly and most importantly, DNA topology influences replication and gene expression. The bacterial chromosome is a highly structured molecule organized into various domains (including macrodomains and replichores) showing varying degrees of gene expression, supercoiling, protein occupancy, and the binding of xenogeneic silencing proteins (e.g., H–NS, histone-like nucleoid-structuring proteins) [14,15,16]. All these physical objects are arranged in a physiological intracellular “ecological topology”, understood as the pattern of interconnections of a molecular network, and based on physical (structural) properties that vary over time (according to growth phases) and should be tightly regulated [17]. For instance, the cell should manage the encounters between replication and transcription machineries, given that conflicts between them can lead to genome instability and reduced fitness [18].

**Figure 1:**
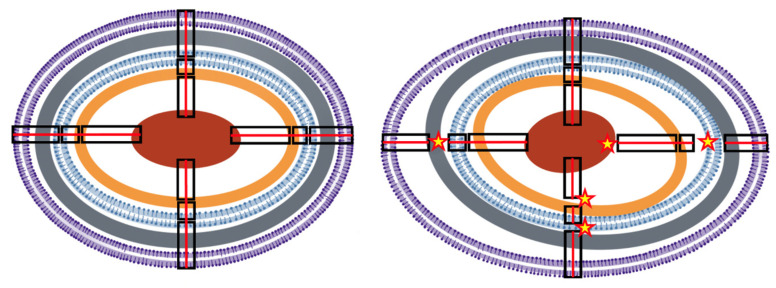
**Conceptual illustration of structural epistasis**. (**Left**) Simplified concentrical structure of a normal Gram-negative bacterium. From the outside to inside, in violet, the bilayer outer membrane (OM); in grey, the peptidoglycan; in blue, the bilayer internal membrane (IM); in brown, the ribosome-dense crown; in red, the nucleoid. Proteins (not represented) are particularly dense among the most external layers. Black rectangles illustrate structural connections across layers (for simplicity only four are depicted), successively, OM–IM, IM–ribosomes, ribosomes–nucleoid. Continuous red lines across the connections illustrate the integrity of the structure. Physiological changes are not expected to eliminate the concentrical structure, as during division, new centers are created. (**Right**) A distorted cellular structure, resulting from exposure to stressful conditions, where the layers are losing their normal connectivity (stars) producing wider spaces in some areas and narrower spaces in others. The result of the architectonical distortion influences the relative molecular interactions (interrupted red lines), changing the local protein densities and interactions (see Figure 2).

In turn, healthy topology contributes to molecular (e.g., rRNA, proteins, nutrients) mobility and interaction, at times mediated by particular compounds, such as histone-like nucleoid-structuring H–NS proteins, which interact with both proteins and DNA [16]. The bacterial cell therefore exhibits a well-controlled spatial organization, with particular degrees of flexibility (adaptive organization) that are beginning to be explored [19,20]. There are precedents for such a type of exploration in the morphogenetic studies of the past century. In 1952, Alan Turing (the godfather of modern computing) proposed that biological morphogenesis could be explained by an stochastic activator–inhibitory system, that gives rise to particular organizational patterns, with modern modeling studies showing the plausibility of this approach [21]. In the following section, a succinct description of the architectural components of the bacterial cell is presented (Figure 1).

## 2. The Molecular Components Involved in Structural Epistasis

**Bacterial cell envelopes:** The complex molecular structure of the bacterial cell envelope, consisting of the inner membrane, the peptidoglycan (PG) sacculus, and the external membrane, is the reactive interphase of the bacterial cell with the environment, and is therefore critical for the bacterial mode of life [22]. The inner membrane (IM, cytoplasmic membrane) in bacteria (such as *E. coli*) has a bilayer structure mostly composed of alpha-helical proteins and phospholipids. Membrane lipids have a critical architectural value, given that small changes in the lipid acyl chains or head groups alter lipids packaging, assuring the architectural robustness when the cell is confronted by environmental changes, including antibiotic exposure [23,24]. The outer membrane (OM, external to the cell wall) is also formed by a bilayer, but also has an asymmetrical structure, with phospholipids and mostly beta-barrel lipoproteins in the inner layer (with more than 100 types in *E. coli*). Lpp, one of these lipoproteins, is the most abundant protein in *E. coli* and is covalently attached to the peptidoglycan, providing a critical connection between the OM and the cell wall [25]. In the OM layer, there is an external dense lipopolysaccharide (LPS) composed of an LPS core, as well as extended polysaccharide chains stabilized by divalent cations (Mg^2+^, Ca^2+^) forming the highly variable O-antigen. This layer also includes transmembrane proteins organized in trimers to form cylinders (outer membrane proteins, OMPs), which can have functional ectodomains with enzymatic activity (e.g., protease in OmpT), but are essentially involved in water and nutrient uptake, and the export of waste products. The biogenesis and integrity of the vital OM requires a constant supply of locally recruited (secreted) OMPs and lipoproteins. Most secretions occur through the Sec translocon, where unfolded OMP polypeptides are delivered to the beta-barrel assembly machine (in turn composed of OMPs and lipoproteins) by periplasmic chaperones, for insertion into the OM. Protein–protein interactions produce a spatiotemporal patterning of the OM into micro-domains, and are the basis of beta-barrel protein turnover [26].

In between the outer and inner membranes, there is a more rigid physical structure, the peptidoglycan sacculus, which is essential for providing the cell shape and volume. The various shapes during the growth phases are ensured by the modularity of the core components of the peptidoglycan synthesis [26]. The peptidoglycan molecular network is formed by sequential chains of the disaccharide N-acetyl glucosamine-N-acetyl muramic acid, which is cross-linked by small pentapeptide chains [27,28]. The space between the IM and OM is known as the “periplasmic space” or simply the “periplasm”, and is an aqueous space extremely rich in proteins and has a higher viscosity than the cytoplasm [29]. An important aspect of the membranes is the physical–structural relationship among the IM, PG and OM, constituting a unified network, which obviously influences the periplasm. As stated earlier, there are membrane adhesion sites joining the IM and OM, such as tripartite efflux pumps [30], and other cross-envelope structures, such as flagella machinery and type III secretion systems.

**Envelope-associated protein-rich peripheral cytoplasm:** The vital multilayered cell envelope determines the other weakly structured molecular layers within the bacterial cytoplasm (protein-rich rings, ribosomes crown) (Figure 1). In fact, almost a half of the total bacterial proteome associates with the bacterial cell envelope [31], constituting a complex proteomic sacculus. It is to be noticed that at least 25% of the total cell proteome is bound and interacts with the IM, frequently under the form of structural oligomeric complexes. Many of these proteins are essential to maintain bacterial life [32].

**Envelope-associated ribosomes-rich peripheral cytoplasm:** In total, there are approximately 50,000 ribosomes per cell, although this number greatly depends on the physiological state (i.e., feast or famine) [33]. There is a crown of ribosomes (the so-called protein factory) bound or in close vicinity (within 50 nm) to the IM, which is compatible with the “transertion hypothesis” (see next section) [33,34,35]. In this ribosome-rich compartment, half of the total osmolality (depending on the number of osmotically active biopolymers per volume unit) is due to ribosomal particles, producing a substantial excluded volume effect that influences protein diffusion [36]. By contrast, the density of ribosomes is low in the cytoplasmic spaces near the bacterial nucleoid [37]. Ribosome density is also affected by changes in their shape and volume, not only as a result of synthesis and degradation (recycling), but also through the polymerization of 70S ribosomes into inactive 100S ribosomes, which allows bacteria to hibernate during stress periods [38].

**Nucleic acid structures in the bacterial cytoplasm:** The bacterial nucleoid is composed of a (most frequently) circular DNA chromosome, floating in the cytosol, but forming in a distinct cell pseudo-compartment that occupies 10–20% of the bacterial cell volume [39,40]. The chromosome has a complex and functionally efficient topology, with plectonemic wound loops of DNA, more or less tightly coiled (positively or negatively supercoiled) and forming approximately 500 supercoiled domains. The multiplicity of the domains limits general damages and facilitates repair processes or relaxation of a single domain without consequences of the superhelicity of other DNA regions. Proteins, particularly topoisomerases, ensure the maintenance, relaxation and restoration of this supercoiled structure, allowing the large RNA polymerase complex to progress along the individual helical turns of DNA. Histone-like proteins, such as the inversion stimulation protein (Fis), the integration host factor (IHF), the heat stable nucleoid-structuring protein (H–NS) and the heat-unstable protein (HU), also contribute to the nucleoid dynamics. During chromosomal segregation, structural maintenance of chromosome (SMC) protein complexes ensure the nucleoid’s architectural preservation (“the choreography”) [41]. Interestingly, the nucleoid might interact with the bacterial envelopes, mostly to fulfill the suggested coupled transcription and translation (CTT) process of the membrane proteins (transertion), which means that the ribosomes initiate the translation of mRNAs, of which the transcription from DNA has not yet concluded, giving rise to “RNA polymerase-.mRNA-ribosome” complexes. The involved gene loci (uracil richness appears to characterize membrane-traversing domains) migrate from the nucleoid complex topology to the vicinity of the inner membrane, and thus become exposed to the ribosome-rich crown in the peripheral cytoplasm. During such a process, additional regulatory mechanisms are involved, such as the levels of the alarmone (p)ppGpp [42].

In the case of plasmid DNA, the replication, partition and transfer processes have been proposed to be dependent on the interaction of plasmid DNA with a limited number of membrane structures (domains) [43]; however, the full demonstration of this plausible hypothesis remains elusive [44]. The localization of plasmid DNA in the cell depends on plasmid-encoded partition genes, moving from the mid-cell position to the 1/4 and 3/4 positions at the time of cell division [45].

**Molecular structures in the bacterial cytoplasm fluid:** Unlike eukaryotic cells, bacteria are devoid of a endoplasmic membranaceous reticulum formed by cytoskeletal proteins, which are mostly involved in cellular functions such as protein synthesis, folding, modification, compartmentalization and transport. However, the bacterial cytoplasm has cytoskeletal-like proteins that might be involved in polymerizing activities [46]. Even in the absence of a real cytoplasmic compartmentalization, as it occurs by overexpressing genetically engineered proteins, the bacterial cytoplasmic organization is ensured by constructing protein and nucleic acid scaffolds that form through liquid–liquid phase separation (LLPS), local dynamic membraneless functional condensates that can enrich specific nucleic acid, and protein components [47,48]. In addition, there are bacterial cytoskeletal protein filaments involved in various processes, including cell elongation, cell division (such as the treadmilling protein, tubulin homolog, FtsZ), chromosomal and plasmid segregation, and cell motility [49]. The structure of the bacterial cytoplasm allows for temporal and spatial localization of proteins (“check points”) involved in the growth cycle progression, maintaining a “cell memory” and ensuring the right topological distributions required for division planes, as it occurs in eukaryotic cells [10]. The preservation of molecular crossroad interactions among nucleotides and proteins is certainly critical at the time of bacterial division [50]. In summary, the bacterial cytoplasm is indeed a crowded microenvironment where numerous potential physical interactions among molecules, macromolecules and protein condensates occur, facilitating structural epistasis.

## 3. The Bacterial Cell Molecular Architecture and Shape Is Altered by Antibiotic Exposure

**Figure 2:**
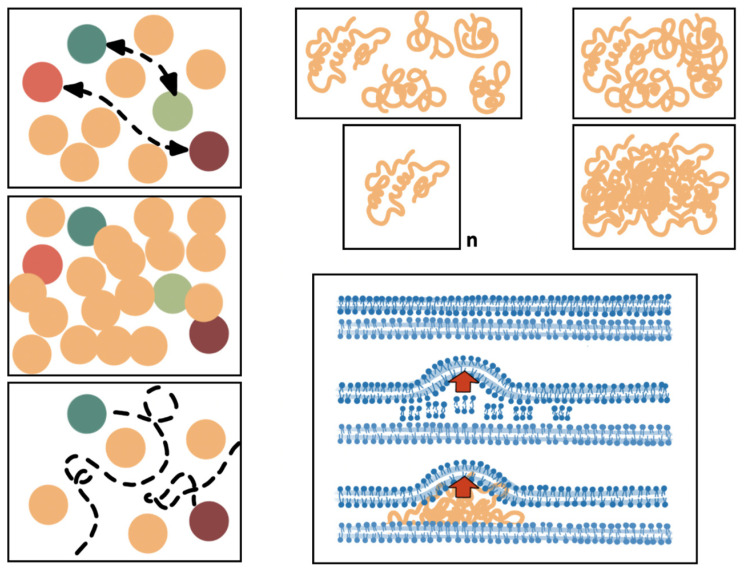
**Effect of structural distortion in the protein–protein and protein–OM interactions**. The **upper left panel** shows a normal density of the proteins (ovals) in a structured space; functional connections (double-headed broken arrows) between particular proteins (light–dark red and light–dark green) occur normally. If the protein density in very high (**mid left panel**), these interactions might be prevented. On the contrary, if the density is too low (**lower left panel**), the connections cannot be established. The **upper right panels** illustrate the 3D folding of proteins (orange crumpled lines). If the protein density is increased, the proteins might interact and eventually change their shape non-specifically. Eventually, conglomerates of the same protein might occur, producing protein inclusion bodies. The **lower right panel** exemplifies the distortion of the normal topology of the OM (up in the panel) due to the overproduction of membrane components (**middle panel**) or the location of a protein inclusion body (e.g., an hyperproduced protein, such as a beta-lactamase). In both cases, the distortion of the OM (red arrow) might alter the location/interaction of particular proteins (including porins, specific receptors, and pumps proteins, not represented for simplicity), which results in altered phenotypes.

Aminoglycosides and fluoroquinolones induce cytoplasmic condensation resulting from cellular membrane damage, with changes in lipid composition and outflow of the cytoplasmic content, and the separation of the OM and IM (larger periplasmic space), ultimately resulting in the release of reactive oxygen species [51,52]. Cytoplasmic condensation favors macromolecule compactification and eventually structural interactions (Figure 2), resulting in either positive (favoring functional enzyme clustering) or negative (by the aggregation of interfering proteins in these functional clusters) epistasis [53,54,55]. In addition, antibiotics that inhibit transcription (as rifamycins) or ribosomal protein synthesis (as chloramphenicol), alter the spatial organization of the bacterial chromosome and ribosomal particles, possibly as a result of changes in cytoplasmic crowding density [56,57]. Consequently, there are changes in intracellular protein and nucleic acid mobility [58], influencing unspecific (resulting from Brownian motion) and specific associations between molecules, such as within multiprotein complexes. Proteins diffuse to reach their functional target locations (Figure 2). Protein and nucleic acids motility and diffusion are critical in exponential growth: their impairment, resulting in diminished intracellular dynamics, could be a reason for the faster antimicrobial effect in fast-growing cells [59], perhaps synergistically with the increased amount of such target structures accessible for antibiotic action.

Due to their specific mechanisms of action, numerous antibiotics affect the architecture and shape of bacterial cells, modifying their normal physical interactions among molecules. Typically, the subinhibitory action of various beta-lactams results (for instance in *E. coli*) in cell elongation or filamentation, as what happens during ampicillin exposure; or cellular rounding, blebbing and dimpling, as during carbapenems challenge [60], which is due to the inhibition of particular penicillin-binding proteins (PBPs) involved in the cell wall synthesis. PBP3 inhibition (as in aztreonam exposure) produces elongated cells, while PBP2 inhibition (as in mecillinam exposure) results in spherical cells [61]. Changes in shapes can also be due to the effect of stress responses; for instance, DNA-targeting antibiotics induce an SOS response involving elongation [62,63,64]. In certain cases, the mechanism of action provokes adaptive response, modifying the cell size and shape; for instance, if the number of active ribosomes is reduced by ribosome-targeting drugs, there is a compensatory over-synthesis of ribosomes, and the cells invest in growth rather than in replication, which results in smaller cells [65]. Protein synthesis inhibition could result in a disbalance in the number of proteins and protein-nucleic acid interactions, thereby deeply affecting bacterial fitness. This disbalance can cause a disturbance in protein and mRNA content that propagates through genetic networks, thereby altering interactions between genes. For instance, microbial cells must coordinate gene expression with cell size and shape [66], and this coordination is essential for phenotypes that affect local fitness, such as motility [67]. Disturbing this coordination has significant effects on cellular phenotypes.

Any change in the cell shape requires an expansion or constriction of the membrane layers (and the volume of the periplasm), involving a quantitative change in their molecular components, or the distance between them, and therefore in their physical interaction. The concept of structural epistasis is applied here to discuss whether these changes might have functional consequences beyond those resulting from classical epistasis, including altered gene expression [68,69]. Protein folding, and hence protein functionality and connectivity, can be altered by membrane molecular changes in the lipid bilayer composition, particularly if influencing the systems (translocons, insertases and chaperones) assuring a correct folding in the membrane [70]. Interestingly, biophysical forces that affect the molecular topology of bacterial cells are still active during cell death; for instance, nanotubes, resulting from the cannibalization of the disintegrated cell membrane, are a post-mortem manifestation [71].

## 4. The Bacterial Cell Architecture and Shape Is Altered by Antibiotic Resistance

Antibiotics frequently promote (or select for) a few phenotypic variants in the population (known as “persisters”) that tolerate antibiotic action at the expense of changing the cellular architecture, resulting in altered cell shapes. In *E. coli* tolerating ampicillin exposure, the persisters are frequently smaller and the cells are swollen, altering physical molecular interactions by changing elasticity or surface-to-volume ratio [72]. In *Staphylococcus aureus*, antibiotic persisters are usually recognized as “small colony variants”, frequently changing the envelope (thicker cell walls) and, correspondingly, a denser granulation in the peripheral cytoplasm, or have branched or multiple cross walls, which are sometimes defective [73]. In addition, exposure to bacteriostatic agents, such as macrolides and chloramphenicol, results in small colony variants [74]. Given that it also occurs in pH-driven transitions, bacterial dormancy or persistence could possibly be due to a transition of the cytoplasm from a fluid to a gel-solid-like state [75,76].

Changes in cell shape induced by antibiotic action have real consequences for the molecular mobility inside the cells (and consequently metabolic activity), which has been tested by using the intracellular diffusion of green fluorescent protein (GFP) in the presence of antibiotics [60,77,78]. The hyperexpression of resistance mechanisms might also affect bacterial architecture. In general, protein hyperexpression leads to the formation of small colony variants and reduces bacterial growth, as was signaled in recombinant strains modified to hyperproduce (for instance using strong promoters) proteins for chemical or pharma industries. To address this problem, the shape of the bacteria should be modified (through morphology engineering) [79]. High levels of TEM-1 beta-lactamase expression results in sequestration of the mature excreted polypeptide in insoluble protein aggregates (inclusion bodies) located within the periplasmic space. Given that correct protein folding is altered in these aggregates, the function is lost [80]. The constitutive hyperexpression of the AmpC-type beta-lactamase might also have a deleterious effect on cellular fitness [81,82]. Studies on *Salmonella*, the only *Enterobacteriaceae* that does not possess AmpC, have shown that the acquisition of exogenous AmpC causes changes in cellular morphology, with longer cells indicating an effect on septation, and produces small changes in the structure of the peptidoglycan. These effects fully abolish *Salmonella Typhimurium* viability, unless *ampC* expression is under control of its regulator, AmpR [81]. A similar effect of a severe fitness cost occurs in *Pseudomonas aeruginosa* [82]. The metallo-carbapenemase NDM-1 is related with lipoproteins of the OM and can be packaged into the periplasmic space by physical interactions with the OM layer, determining “envelope stress” and facilitating its extrusion inside membrane vesicles [83]. MDR efflux pump hyperexpression should also physically disturb the anatomical and functional structure of the cell envelope, with consequent effects of an altered cell physiology. However, this structural disturbance is still an underexplored area. The fact that particular *Stenotrophomonas maltophilia* resistant mutants present a reduced cell size and a lower plate efficiency might shed light the direction of future studies [84].

Nevertheless, efflux pump overexpression modifies the activity of other unrelated cell machineries, thereby providing an example of structural epistasis. This is the case for MexEF-OprN in the *Pseudomonas aeruginosa*, of which the overexpression increases oxygen respiration, modifies the intracellular pH and triggers the activation of the nitrate respiratory pathway [85]. Notably MexEF-OprN overexpression is associated with a reduction in type III secretion [86], and type III secretion requires significant activity of the proton motive force [87]. Whether MexEF-OprN overexpression inactivates type III secretion by modifying the proton motive force, thereby impeding the simultaneous induction of these two energetically costly cell machineries, and if the reduction in fitness is also influenced by less efficient basic metabolic pathways (as energy in invested in overexpression of the secreted protein), are questions that remain unanswered.

The conjugative process of plasmids (which eventually encode antibiotic resistance) might produce (probably small) alterations in the bacterial architecture of the donor cell, because of the relaxosome protein complex (docked to the Type IV secretion system) and the entire conjugative apparatus, forming an envelope structure bridging the IM and the OM [88]. In the donor and recipient cells, the exit and the entry of the ssDNA plasmid triggers the local recruitment of Ssb (single-strand binding protein) molecules and the formation of membrane conjugative foci, which are apparently located at specific membrane positions, possibly related to the density and stability of the outer membrane protein OmpA, a beta-barrel porin collaborating in the process [89]. Eventually, mobile genetic elements (preferentially small plasmids) might contribute to increasing particular gene dosages, and thus gene dosage toxicity is based on abnormal protein abundance levels. Dihydrofolate reductase overexpression in *E. coli* causes a metabolic imbalance, reducing bacterial fitness [90].

## 5. Cell Architecture and Cell Size Influences Antibiotic Effects

Any change in the cell shape requires an expansion or constriction of the membrane layers (and the periplasm volume), which involves a quantitative change in their molecular components or in the distance between them, and therefore their physical interaction. The concept of structural epistasis is applied here to discuss whether these changes might have functional consequences, including the susceptibility to antimicrobial action [66]. Quantitative modeling has shown that bacteria might adapt (reduce their susceptibility) to antibiotic challenges by reducing the surface-to-volume (S/V) ratio, and consequently, the antibiotic influx and intracellular concentration. In certain cases, however, increasing this ratio might provide an increase in antibiotic efflux rate, and thereby reduced susceptibility. Moreover, the concentration of membrane-associated antibiotics is reduced [65]. In fact, most tested antibiotics decrease the S/V ratio. Such a reduction also decreases nutrient uptake, slowing the bacterial metabolism and consequently the antibiotic action [91]. As expected, the morphological cell response to membrane-targeting and membrane-transport-targeting antibiotics is a reduction in the cell surface area. Other effects of the S/V can be considered secondary to the bacterial adaptation to drug action; for instance, the inhibition of translation by ribosome-targeting antibiotics (as chloramphenicol) is compensated by a higher ribosomal biosynthesis, leading to the predominance of growth versus replication, with the consequences of an increased cell volume [91].

Membrane changes are not necessarily global. Different bacterial processes are confined to membrane microdomains that are similar to lipid eukaryotic cell lipid rafts, which accumulate multimeric protein complexes favoring their oligomerization. One of these proteins, PBP2a, causes beta-lactam resistance in methicillin-resistant *Staphylococcus aureus* (MRSA). Notably, the disruption of these membrane microdomains with available drugs (such as statins, regularly used for treating hypercholesterolemia) interferes with PBP2a oligomerization, resulting in MRSA infections that are treatable with penicillin [92]. This example shows that structural alterations in a cellular element (the cell membrane) impedes the structural changes (oligomerization) required for the activity of a wide-spread antibiotic resistance gene.

In addition, bacteria readily alter their shape in response to non-antibiotic cues. For instance, during urinary tract infections, *E. coli* produces long multi-nucleated filaments in response to a still unknown urine component [93]. In addition to decreasing the S/V ratio, filamentation has numerous consequences for the bacterial lifestyle. For instance, filamented bacteria are less likely to be attacked by phagocytes [94], and thanks to the extra body-mass, filamented bacteria might have an improved ability to resist shear forces in the bladder and adhere to the epithelium [95,96]. Beyond the urinary tract, filamentation allows intracellular bacteria to spread among host cells [97] and might promote the evolution of antibiotic resistance [98], which provides an excellent example of how physical changes in cell structure can shape numerous, non-related phenotypes.

## 6. Spatial Cell Biology, Molecular Interactome, and Antibiotic Actions

The main concept suggested in this review is that antibiotics and antibiotic resistance modify the intracellular “molecular ecology” [99], particularly the structural (membranes, cytoskeleton) and functional (proteins, nucleic acids) components that determine the spatial bacterial cell biology. More than 1300 unique proteins have been identified in *E. coli* [98]. Most functional molecules should move within the cell to localize their target sites, which may change according to the cell phase and stress conditions. In the case of proteins, localization is frequently determined by binding to another previously localized protein that serves to target a particular functional site [100,101]. Binding to the signaling protein can be expected to be hampered by molecular crowding and loss of cytoplasmic fluidity, and might produce abnormalities if the right site is not reached by the guiding protein. Moreover, the number of potential protein–protein interactions (PPIs) inside the cell is huge: in *E. coli*, there are several hundreds of macromolecular complexes, and the total PPIs in these groups probably exceed 10,000. However, many PPIs are not in complexes or are difficult to detect because they are transient [102]. However, transient interactions between proteins determine the “quinary structure” (meta-PPI in multimeric proteins), which provides important features regarding intracellular organization and compartmentalization [103]. In fact, an increase in nonspecific interactions (e.g., mediated by protein membrane charge) in the crowded cellular space should compete with specific interactions and interfere with cellular functions [103,104]. It could be expected that forced PPIs derived from alterations in protein density following cellular architectural alterations might result in aggregation, misfolding, and functional impairment (Figure 2), but this is still an open field for further research. Bioinformatics techniques based on deep learning are expected to be applied soon to predict PPIs (also protein domain–domain and protein–RNA interactions), and to ascertain their possible consequences in the presence of changes in architectural and physical cellular damage [104,105].

## 7. Conclusions

We are far from understanding the effects of the alterations in the balance and physical (structural) molecular interactions among the various gene products, which should produce functional abnormalities in the bacterial cell, eventually pushing cells to death. Bacterial death, and any death, is the result of cellular architectural disorganization [106]. Unfortunately, establishing a catalog of consequences of the modifications in the interactome that result from changes in the cellular shape and architecture, caused by either antimicrobial exposure or the expression of antibiotic resistance, is not an easy task. We expect, however, that these changes should necessarily occur. How the architecture of bacterial cells has evolved, so that differing lineages exposed to different environments and requiring different adaptive needs have different cellular shapes and subcellular spatial compartmentalization, and this is a starting field of research [27]. The acquisition of this knowledge, in combination with the establishment of a physical interactive landscape of various intracellular interactions under differing conditions of growth and stress, is certainly needed. Such knowledge could contribute to the better understanding of the mechanism of action of antimicrobials [107], the mechanisms governing the laws of associations between drugs, and the fitness costs of the acquisition of antibiotic resistance determinants.

## Data Availability

There are no available datasets analyzed or generated during the study.
